# Study on Basalt Fiber-Reinforced Lunar Regolith Simulant Geopolymer: Experiment and Constitutive Model

**DOI:** 10.3390/ma19143037

**Published:** 2026-07-14

**Authors:** Jianghuai Zhan, Lepeng Huang, Ziheng Ding, Fei Wang, Shuai Li, Xuanyi Xue, Jianmin Hua

**Affiliations:** 1 School of Civil Engineering, Chongqing University, Chongqing 400045, China; 2 State Key Laboratory of Safety and Resilience of Civil Engineering in Mountain Area, Chongqing 400045, China; 3 Department of Civil Engineering, The University of Hong Kong, Pokfulam Road, Hong Kong, China

**Keywords:** lunar regolith simulant, basalt fiber length, compressive strength, flexural strength, stress–strain constitutive model, microstructure

## Abstract

Lunar regolith simulant (LRS) geopolymers are promising construction materials for lunar in situ resource utilization, but their brittle behavior and limited crack resistance restrict their structural applications. This study investigated the effect of basalt fiber length on the mechanical properties, failure modes, stress–strain behavior, constitutive relationship, and microstructure of CQU-1 LRS geopolymers. Basalt fiber-reinforced LRS geopolymers were prepared under weak alkali activation and high-temperature curing at 80 °C. The basalt fiber content was fixed at 0.1%, and six fiber lengths of 0, 6, 9, 12, 15, and 18 mm were considered. Compressive and flexural tests were conducted after curing for 1 d and 7 d, and the normalized stress–strain curves were fitted using the Saenz L.P., Carreira D.J., and Zhenhai Guo models. The results showed that basalt fiber length significantly affected the mechanical performance of LRS geopolymers. An appropriate fiber length improved strength, stiffness, ductility, and post-peak load-bearing capacity, whereas excessively short or long fibers weakened the reinforcing effect. The 15 mm fiber group exhibited the best overall performance. After curing for 1 d, its compressive strength reached 2.23 MPa, 49.7% higher than that of the control group, and its elastic modulus increased approximately 2.5-fold. After curing for 7 d, its compressive strength reached 13.44 MPa, 32.0% higher than that of the control group. The Zhenhai Guo model provided the best fit for the stress–strain curves. SEM-EDS analysis showed that basalt fibers improved interfacial bonding and promoted gel enrichment near the fiber–matrix interface. Overall, 15 mm was recommended as the optimal basalt fiber length for CQU-1 LRS geopolymers under the conditions used in this study.

## 1. Introduction

With the rapid development of deep-space exploration technology, the construction of lunar research stations, lunar roads, landing platforms, protective structures, and habitation modules has gradually become an important part of future long-term lunar residence missions. However, if traditional construction materials were to be transported entirely from Earth, the process would not only be extremely expensive but would also be limited by launch capacity and mission duration, making it difficult to meet the demand for large-scale lunar construction. Therefore, using lunar surface in situ resources to prepare construction materials is considered an important approach for achieving sustainable lunar base construction [1,2].

In recent years, extensive research has been conducted on lunar in situ construction and extraterrestrial construction material systems. Li et al. [3] proposed an extraterrestrial bioconstruction technology system and emphasized the importance of the coordinated development of in situ resource utilization, biomanufacturing, and intelligent construction for long-term deep-space residence. Furthermore, Li et al. [4] investigated the mechanical–energy evolution and damage mechanisms of laser powder bed fusion-formed lunar regolith simulant and pointed out that energy dissipation and crack propagation were critical for evaluating the engineering applicability of lunar regolith materials. Zhou et al. [5] used digital image correlation and high-speed photography to reveal the local deformation, crack propagation, and failure process of vacuum-sintered lunar regolith simulant (LRS). These studies indicated that research on lunar regolith materials had gradually shifted from single strength evaluation to comprehensive analysis of forming methods, damage evolution, deformation capacity, and service reliability.

Lunar regolith is widely distributed on the lunar surface and is rich in oxides such as SiO_2_, Al_2_O_3_, CaO, and Fe_2_O_3_, showing potential as a geopolymer precursor [6]. Geopolymers are inorganic cementitious materials formed by the alkali activation of aluminosilicate raw materials and have advantages such as low carbon emissions, early strength development, and high-temperature resistance [7]. Therefore, lunar regolith-based geopolymers are considered important candidate materials for lunar in situ construction [8]. Because real lunar regolith samples are scarce, lunar regolith simulants are commonly used for ground-based verification. Zheng et al. [2] reviewed the application potential of lunar regolith geopolymer concrete in lunar base construction and pointed out that lunar vacuum and thermal cycling affected its performance stability. Mills et al. [7] demonstrated that both lunar and Martian regolith simulants could be used to prepare geopolymers through alkali activation. Zhou et al. [9] found that reasonable control of alkali content and mix proportion could improve the strength of BH-1 LRS geopolymers. Xiong et al. [10] indicated that extreme temperatures affected the structure and mechanical properties of LRS geopolymers. Chen et al. [11] showed that particle size, liquid composition, and alkali content played important roles in the strength development of geopolymer materials for lunar road construction.

For CQU-1 LRS geopolymers, Lu et al. [12] investigated their short-age mechanical properties and indicated that sodium silicate solution effectively activated CQU-1 LRS to form geopolymer materials with a certain load-bearing capacity. Zhan et al. [13] further studied the effects of curing temperature on the early-age mechanical properties and microstructure of CQU-1 LRS geopolymers and found that high-temperature curing promoted the alkali activation reaction and the formation of gel products, thereby improving the early-age strength of the materials. These studies showed that LRS geopolymers have promising application prospects, but their performance is still affected by factors such as raw material composition, activator type, alkali content, and curing regime. Recent studies on lunar regolith-based geopolymers have increasingly focused on alkaline activation, curing conditions, particle size regulation, and mechanical performance improvement. These studies confirmed the feasibility of preparing cementitious materials from LRS, but also showed that their strength development and deformation capacity are strongly affected by precursor mineralogy, activator chemistry, and curing regime. Meanwhile, fiber-reinforced geopolymer composites have been widely investigated to improve tensile resistance, crack control, and post-cracking toughness. Basalt, carbon, glass, and polymer fibers have been reported to enhance the ductility and fracture resistance of geopolymer matrices through bridging and pull-out mechanisms. In addition, constitutive modeling of geopolymer materials has attracted increasing attention because stress–strain models are necessary for mechanical prediction and structural design. However, most existing constitutive models were originally developed for ordinary concrete, and their applicability to LRS geopolymers remains unclear. Therefore, the combined investigation of basalt fiber length, mechanical behavior, and constitutive model fitting is necessary for advancing the design of extraterrestrial construction materials and ISRU-based structural systems.

Although LRS geopolymers have a certain load-bearing capacity, they are inorganic cementitious materials and still have several limitations, such as insufficient tensile and flexural properties, obvious brittle failure, rapid crack propagation, and limited post-peak load-bearing capacity. For lunar structural materials, good crack resistance, toughness, deformation capacity, and service stability are required, in addition to compressive strength. Fiber reinforcement could provide bridging, tensile restraint, and crack-arresting effects in the matrix, and is an effective method for improving the mechanical properties and toughness of brittle cementitious materials [14,15]. Among different fibers, basalt fiber has high tensile strength, a high elastic modulus, high-temperature resistance, and good compatibility with silicate-based matrices, making it a potential reinforcing material for LRS geopolymers. However, basalt fibers may suffer from surface corrosion or strength degradation in alkaline environments, especially under long-term exposure or high alkalinity. Therefore, the reinforcing effect of basalt fibers in alkali-activated systems should be evaluated together with their alkali stability [16]. Kjøniksen et al. [14] demonstrated that basalt fiber improved the crack control ability and mechanical properties of LRS geopolymers. Debbarma et al. [15] found that basalt fiber enhanced the flexural performance of LRS geopolymers and could act as a nucleation site for gel products, thereby improving the microstructure. Zhan et al. [1] investigated the effect of basalt fiber content on the static mechanical properties of CQU-1 LRS geopolymers and showed that an appropriate amount of basalt fiber improved the ductility and crack resistance of the materials.

However, existing studies mainly focused on LRS type, activator composition, curing regime, forming method, and fiber content, while the effect of basalt fiber length, a key geometric parameter, remains insufficiently investigated. Fiber length directly affects anchorage, crack bridging, and load transfer in the geopolymer matrix. Fibers that are too short cannot effectively arrest cracks, whereas excessively long fibers tend to cause poor dispersion, agglomeration, and pore defects. Therefore, the mechanism by which basalt fiber length affects the mechanical properties, failure behavior, stress–strain relationship, and microstructure of LRS geopolymers still needs to be clarified. Based on this, CQU-1 LRS was used as the matrix material in this study, and basalt fiber-reinforced LRS geopolymers were prepared under weak alkali activation and high-temperature curing at 80 °C. Based on previous results, the basalt fiber content was fixed at 0.1%. Fiber length and curing age were selected as the main variables, and six fiber lengths, namely 0, 6, 9, 12, 15, and 18 mm, were considered. The performance evolution of the materials was investigated after curing ages of 1 d and 7 d. Compressive tests, flexural tests, and stress–strain curve measurements were conducted to analyze the effects of different fiber lengths on compressive strength, flexural strength, failure mode, elastic modulus, ultimate strain, and post-peak load-bearing capacity. Meanwhile, the Saenz L.P. model, Carreira D.J. model, and Zhenhai Guo models were used to fit the normalized stress–strain curves, and the applicability of different constitutive models was evaluated. SEM-EDS and TG-DTG tests were also conducted to reveal the reinforcing mechanism of basalt fibers. The scientific contribution of this study lies in clarifying the role of basalt fiber length in regulating the mechanical behavior and constitutive response of CQU-1 LRS geopolymers. Previous studies mainly focused on LRS type, activator composition, curing regime, forming method, and fiber content, whereas the influence of fiber length on crack-bridging efficiency, load transfer, post-peak behavior, and stress–strain constitutive relationship remains insufficiently understood. In this study, basalt fiber length was selected as the key variable, and its effects on compressive strength, flexural strength, failure mode, elastic modulus, peak strain, and post-peak load-bearing capacity were systematically evaluated. By combining mechanical tests, constitutive model fitting, and SEM-EDS analysis, this work provides new insight into the length-dependent reinforcement mechanism of basalt fibers in LRS geopolymers and offers a reference for the mechanical design of lunar in situ construction materials.

## 2. Materials and Experiment Methods

### 2.1. Materials

**Figure 1:**
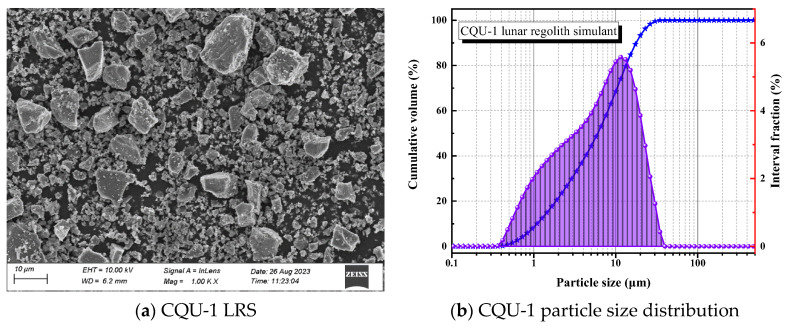
SEM comparison of CQU-1 LRS and real lunar regoliths: (**a**) CQU-1 LRS; (**b**) CQU-1 particle size distribution.

The CQU-1 LRS used in this study was the same as that used in Refs. [1,13]. Besides chemical composition, the mineralogical composition of lunar regolith is also critical for its alkaline activation reactivity. According to published mineralogical studies, lunar regolith is mainly composed of plagioclase, pyroxene, olivine, ilmenite, and a certain amount of glassy or amorphous phases. Among these phases, crystalline minerals such as plagioclase, pyroxene, and olivine are relatively less reactive under alkaline activation, whereas glassy or amorphous aluminosilicate phases are generally more susceptible to alkaline dissolution and geopolymerization. Therefore, the mineralogical similarity and glassy phase content of CQU-1 LRS should be considered when evaluating its suitability as an aluminosilicate precursor. To clarify its chemical composition, X-ray fluorescence (XRF) analysis was conducted to systematically compare several real lunar regolith and LRS samples, including Chang’E-5 lunar regolith [17], Apollo 14 lunar regolith [18], BH-1 [9], HUST-1 [19], and CQU-1 [20]. The XRF results are shown in Table 1. Both real lunar regolith and LRS contained SiO_2_, TiO_2_, Al_2_O_3_, Fe_2_O_3_, CaO, MgO, Na_2_O, K_2_O, and P_2_O_5_. Therefore, CQU-1 LRS had the basic material conditions required to replace real lunar regolith for geopolymer preparation. The SEM observations in Figure 1 show that CQU-1 LRS was highly consistent with real lunar regolith in terms of particle morphology and surface structure, and its elemental composition also conformed to the typical characteristics of real lunar regolith [21]. Based on the above similarity analysis, CQU-1 LRS was selected for the subsequent experimental analysis in this study. Its particle size distribution is shown in Figure 1b. CQU-1 LRS was prepared from basaltic silico-aluminous raw materials to reproduce the major chemical and mineralogical characteristics of mare-type lunar regolith. These raw materials were selected because basaltic lunar regolith is generally rich in Si-, Al-, Fe-, Mg-, and Ca-bearing minerals, such as plagioclase, pyroxene, olivine, and ilmenite. Therefore, CQU-1 LRS can approximate the oxide composition, particle morphology, and basaltic mineralogical characteristics of real lunar regolith. However, the alkaline activation reactivity of LRS depends strongly on the content of glassy or amorphous phases, because these phases dissolve more readily than crystalline minerals and provide reactive Si and Al species for geopolymer gel formation. The amount of glassy or amorphous phase is particularly important for alkaline activation because it provides more reactive Si and Al species than crystalline minerals. In this study, the glassy phase content of CQU-1 LRS was not quantitatively determined by XRD–Rietveld refinement, selective dissolution, or NMR. Therefore, the exact contribution of the amorphous phase to the geopolymerization reaction could not be directly evaluated. This limitation should be considered when interpreting the relatively low mechanical strength of the prepared LRS geopolymers.

For the preparation of the alkaline activator, a composite solution composed of sodium silicate, sodium hydroxide, and deionized water was used in this study. The initial modulus of the sodium silicate was 3.3, with a SiO_2_ mass fraction of 26.5% and a Na_2_O mass fraction of 8.3%. To adjust the modulus of the silicate system, sodium hydroxide flakes with a purity of no less than 99% were added. Deionized water was used as the reaction medium to avoid interference from impurities during the experiment and to maintain the chemical purity of the system.

The basalt fibers adopted in this study were supplied by Anjie Company, Jiaxing, China, and their main physical properties are summarized in Table 2. Each fiber had a monofilament diameter of 10 μm, with a density ranging from 2.63 to 2.65 g/cm^3^. The elastic modulus and tensile strength were 91–110 GPa and 3000–4800 MPa, respectively.

### 2.2. Experimental Variable Design

Under weak alkali activation and high-temperature curing conditions, cohesive gel polymers could be formed inside the LRS geopolymer, while excessive pores were less likely to be generated. Therefore, in this section, basalt fiber-reinforced CQU-1 LRS geopolymers were prepared using weak alkali activation combined with high-temperature curing at 80 °C. According to previous experimental results, the mechanical properties of the LRS geopolymer were optimal when the basalt fiber content was 0.1%. Therefore, the basalt fiber content was set as 0.1% in this study [1]. It should be noted that fiber length was a key geometric parameter affecting the reinforcement effect and load transfer mechanism. Fibers that were too short could not fully exert their bridging and crack-arresting effects, whereas excessively long fibers tended to be unevenly dispersed in the matrix and introduced additional defects. Therefore, it was essential to systematically investigate the regulatory mechanism of fiber length on the mechanical properties of LRS geopolymers. In this section, basalt fiber length (6, 9, 12, 15, and 18 mm) and high-temperature curing age were selected as the key variables. To ensure the reliability of the test results, three parallel specimens were prepared for each group. A total of 36 compressive specimens and 36 flexural specimens were prepared, with 72 specimens in total.

### 2.3. Geopolymer Specimen Preparation

In this study, LRS geopolymer specimens were prepared according to GB/T 17671-2021 [22] and BS EN 196-1:2016 [23]. A weak alkali activation system was adopted, and pure sodium silicate solution was used as the alkaline activator. Its initial modulus was 3.3, the Na_2_O content was 8.3%, and the water-to-binder ratio was set to 0.456. According to the mix proportions shown in Table 3, the LRS raw material was weighed and added to a stainless-steel mixing bowl. Mixing was conducted using a JJ-5 planetary cement mortar mixer (Nanjing Tester Instruments Co., Ltd., Nanjing, China). After the predetermined mass of alkaline activator was added, the mixture was first stirred at a low speed of 140 ± 10 r/min for 2 min and then at a high speed of 285 ± 10 r/min for 3 min. The fresh paste was poured into 40 mm cubic molds and 40 mm × 40 mm × 160 mm prismatic molds, vibrated on a vibrating table to remove entrapped air, leveled, and then covered with polyethylene film to prevent moisture evaporation. The specimens were then transferred to a drying oven for curing and demolded after 24 h.

### 2.4. Mechanical Test Method

The flexural test in this study was conducted using a universal testing machine at a loading rate of 50 N/s. The experimental setup for compressive property and stress–strain curve testing in this study utilized an MTS CMT5105 electronic universal testing machine. During testing, 50 mm adhesive-based strain gauges were securely and uniformly attached to the mid-section of all four lateral surfaces of the specimen in both longitudinal and transverse orientations. These gauges were connected to a TDS-150 static data acquisition system to monitor specimen deformation in real time. The specimen was then positioned on the lower loading platform and subjected to displacement-controlled loading at a rate of 0.15 mm/min until failure occurred and the load-bearing capacity dropped significantly, with load data being recorded by a pressure sensor. To minimize experimental error, an additional displacement transducer was installed at the lower loading end of the testing machine to record the displacement–time history of the specimen via digital acquisition. Compressive strength values were determined by averaging the results from three parallel specimens, while the stress–strain curve selected for analysis was the one closest to the average response. The elastic modulus test was conducted in accordance with the Chinese standard GB/T 50081-2019 [24]. For each group, three parallel specimens were tested, and the reported mechanical properties were calculated as the average values. The error bars in the column charts represent the standard deviations of the three parallel specimens. The elastic modulus of the specimen was calculated using the following formula:

(1) $$E_{c}=\frac{F_{a}-F_{0}}{A}\times \frac{l}{\Delta l}$$

where *E*_c_ is the elastic modulus of the specimen (MPa); *A* is the cross-sectional area of the specimen (mm^2^); *F*_a_ = 0.2 *f*_u_, where *f*_u_ is the ultimate compressive strength (MPa); *F*_0_ = 0.1 *f*_u_, Δ*l* is the deformation of the specimen when loaded to *F*_a_; *l* is the height of the specimen (mm).

## 3. Results and Analysis

### 3.1. Mechanical Property

#### 3.1.1. Compressive Strength and Failure Mode

**Figure 2:**
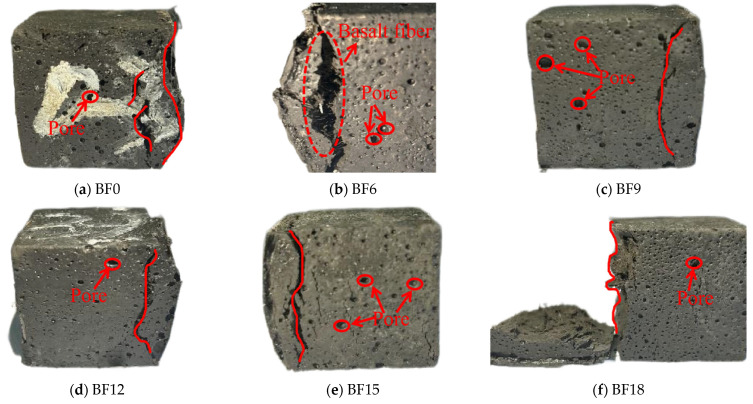
Effect of different fiber lengths on compressive failure modes of LRS geopolymers after curing for 1 d.

Under weak alkali activation and curing at 80 °C for 1 d, the failure modes of LRS geopolymers with different fiber lengths are shown in Figure 2. During compressive failure, all specimens formed a penetrating crack on one side and rapidly lost their load-bearing capacity, indicating an overall brittle failure mode. For the specimens without fibers and those with short fibers (6 mm), large horizontal deformation was observed, and the cracks in the fiber-free specimens were irregular and discontinuous. After basalt fibers were incorporated, the continuity of the penetrating cracks was enhanced, and the horizontal deformation was effectively restrained, indicating that the fibers restricted crack propagation and improved the stress state of the specimens. As the fiber length increased, the crack width first decreased and then increased, suggesting that fibers with an appropriate length provided better crack control. Basalt fibers were observed on the fracture surfaces of all fiber-reinforced specimens, indicating that no obvious macroscopic degradation of basalt fibers was observed under the short-term weak alkali activation and curing conditions used in this study. However, this observation does not imply long-term alkali stability, and further durability tests are required to evaluate basalt fiber degradation in alkaline LRS geopolymer matrices.

**Figure 3:**
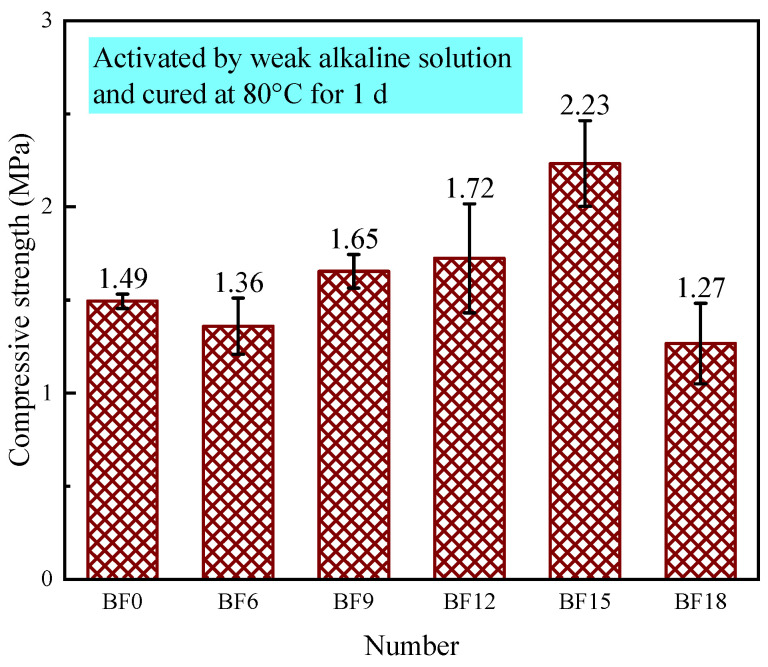
Effect of different fiber lengths on compressive strength of LRS geopolymers after curing for 1 d.

Under weak alkali activation and curing at 80 °C for 1 d, the effect of different fiber lengths on the compressive strength of LRS geopolymers is shown in Figure 3. The compressive strength ranged from 1.27 to 2.23 MPa. Compared with the control group, when the basalt fiber lengths were 6, 9, 12, 15, and 18 mm, the compressive strength of the LRS geopolymers changed by −8.7%, 10.7%, 15.4%, 49.7%, and −14.8%, respectively. With increasing basalt fiber length, the compressive strength of the basalt fiber-reinforced LRS geopolymers first decreased, then increased to a peak value, and finally dropped sharply. The results indicated that, under this curing condition, basalt fibers with an appropriate length significantly improved the compressive strength of LRS geopolymers, whereas fibers that were too short or too long adversely affected their compressive performance. The compressive strength reached its peak when the basalt fiber length was 15 mm.

**Figure 4:**
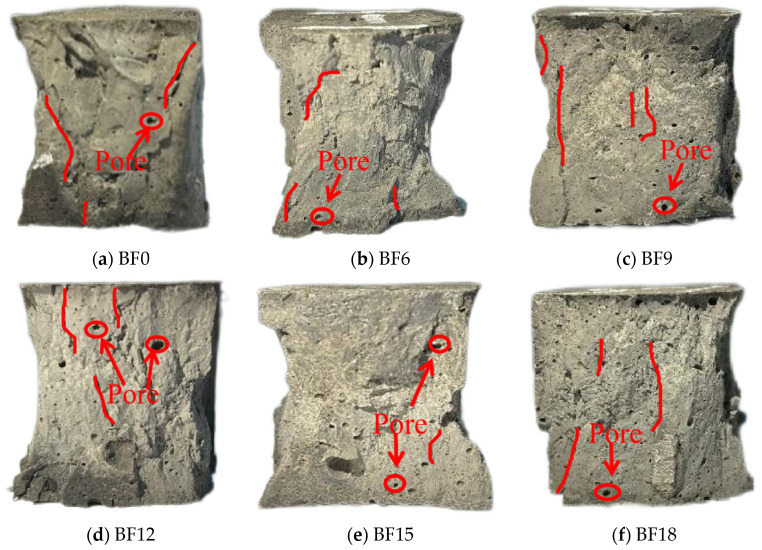
Effect of different fiber lengths on compressive failure modes of LRS geopolymers after curing for 7 d.

Under weak alkali activation and curing at 80 °C for 7 d, the compressive failure modes of LRS geopolymers with different fiber lengths are shown in Figure 4. With increasing curing age, all specimens generally exhibited brittle failure. The specimens reinforced with basalt fibers showed a relatively obvious confinement effect, and their failure morphology was approximately pyramidal. During uniaxial compression, the control group failed rapidly with a crisp sound, and only a small amount of debris peeled off around the specimen. After basalt fibers were incorporated, the failure sound became duller, more fragments peeled off from the surrounding area, and no obvious penetrating cracks appeared. The fiber-reinforced specimens still maintained a certain load-bearing capacity after reaching the peak strain of the control group, indicating that basalt fibers improved the compressive performance and ductility of the core load-bearing structure of the LRS geopolymers. In addition, the fracture surfaces of the specimens turned white, indicating that basalt fibers, LRS, and the weak alkaline solution jointly formed white gel polymers under high-temperature conditions and participated in the load-bearing structural system of the specimens.

**Figure 5:**
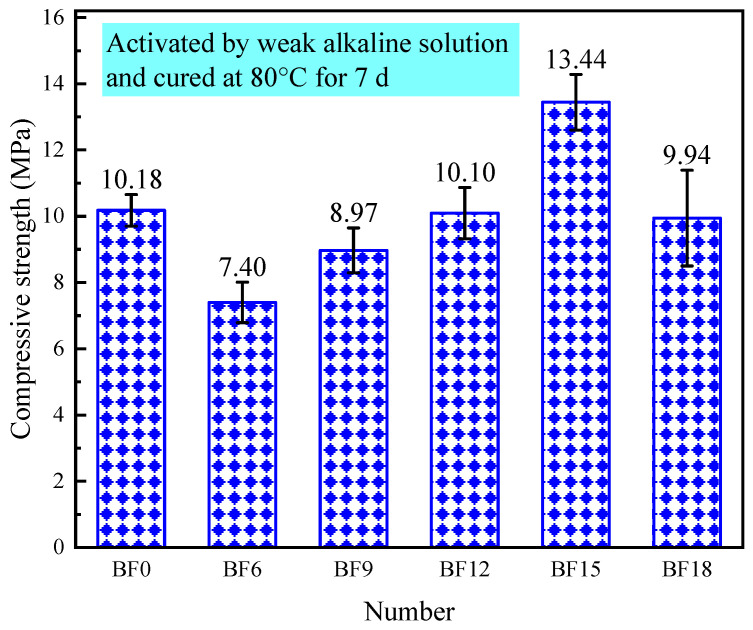
Effect of different fiber lengths on compressive strength of LRS geopolymers after curing for 7 d.

Under weak alkali activation and curing at 80 °C for 7 d, the effect of different fiber lengths on the compressive strength of LRS geopolymers is shown in Figure 5. The compressive strength of the LRS geopolymers ranged from 7.40 to 13.44 MPa. Compared with the control group, when the basalt fiber lengths were 6, 9, 12, 15, and 18 mm, the compressive strength changed by −27.3%, −11.9%, −0.8%, 32.0%, and −2.3%, respectively. As the basalt fiber length increased, the compressive strength generally first decreased, then increased to a peak value, and finally decreased again. Only the 15 mm basalt fiber improved the compressive strength of the LRS geopolymer, whereas the other fiber lengths weakened the compressive performance to varying degrees. These results indicated that, under weak alkali activation and curing at 80 °C for 7 d, basalt fiber length had a significant effect on the compressive performance of LRS geopolymers. An appropriate fiber length promoted strength development, whereas fibers that were too short or too long were unfavorable for compressive performance.

#### 3.1.2. Flexural Strength and Failure Mode

**Figure 6:**
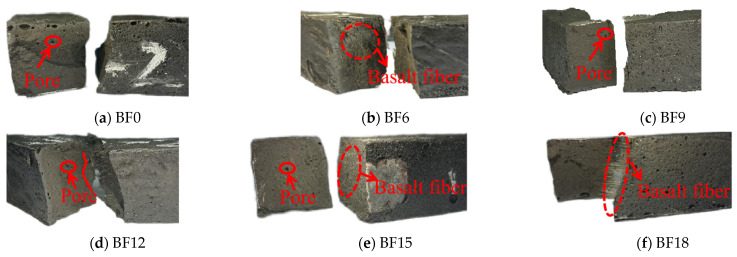
Effect of different fiber lengths on flexural failure modes of LRS geopolymers after curing for 1 d.

Under weak alkali activation and curing at 80 °C for 1 d, the failure modes of LRS geopolymer flexural specimens with different fiber lengths are shown in Figure 6. All specimens failed relatively slowly. After cracks appeared, the specimens did not fracture immediately, and the crack widths were relatively large, indicating an overall ductile failure mode. After basalt fibers were incorporated, the deformation capacity of the specimens was further improved, and they still maintained a certain load-bearing capacity after large deformation, indicating that the fibers bridged cracks and enhanced ductility. As the fiber length increased, the crack width at the bottom of the flexural specimens gradually increased. The fracture surfaces of the specimens appeared black, and protruding basalt fibers were observed on the failure surfaces, indicating that the fibers participated in the flexural load-bearing system and provided reinforcement. Meanwhile, the fracture surfaces were relatively dense and contained few pores, suggesting that basalt fibers did not adversely affect the hardening and formation of the internal structure of the LRS geopolymer under this curing condition.

**Figure 7:**
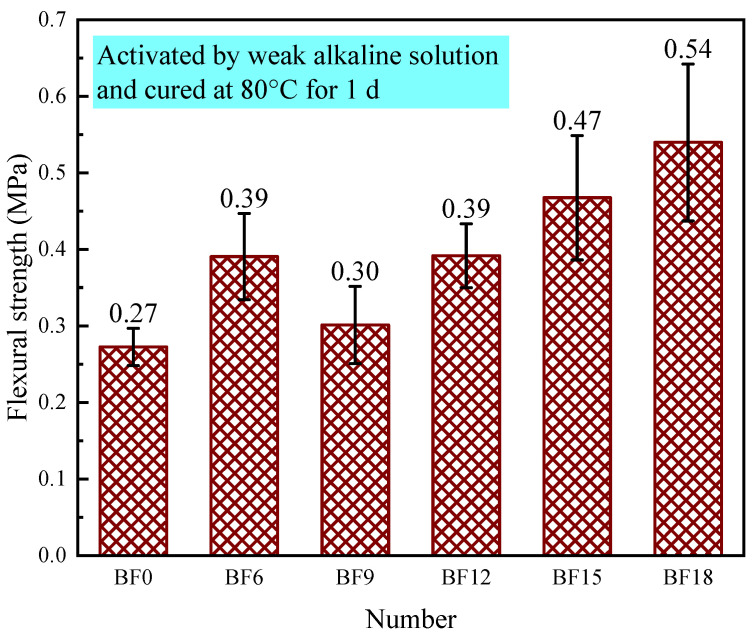
Effect of different fiber lengths on flexural strength of LRS geopolymers after curing for 1 d.

Under weak alkali activation and curing at 80 °C for 1 d, the flexural strength of LRS geopolymers with different fiber lengths is shown in Figure 7. The flexural strength ranged from 0.27 to 0.54 MPa. Compared with the control group, the incorporation of basalt fibers improved the flexural strength of the LRS geopolymers. When the fiber lengths were 6, 9, 12, 15, and 18 mm, the flexural strength increased by 44.4%, 11.1%, 44.4%, 74.1%, and 100%, respectively. As the basalt fiber length increased, the flexural strength generally first increased, then decreased, and then continued to increase, indicating that increasing fiber length was beneficial for improving the flexural performance of LRS geopolymers. The flexural strength reached its peak when the fiber length was 18 mm.

**Figure 8:**
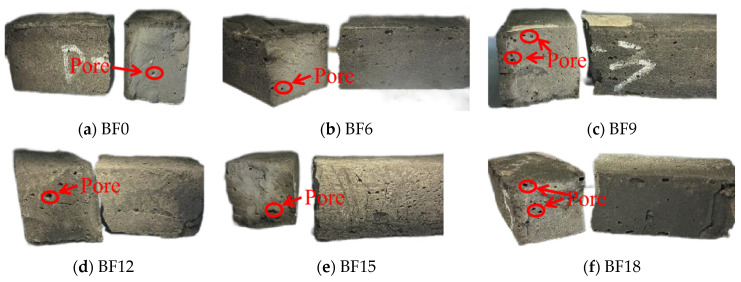
Effect of different fiber lengths on flexural failure modes of LRS geopolymers after curing for 7 d.

Under weak alkali activation and curing at 80 °C for 7 d, the effects of different fiber lengths on the failure modes of LRS geopolymer flexural specimens are shown in Figure 8. All specimens failed rapidly and exhibited small deformation, indicating an overall brittle failure mode. After basalt fibers were incorporated, the deformation capacity of the specimens was improved to some extent, and they still maintained a certain load-bearing capacity after reaching the ultimate strain of the control group. After cracks appeared, the control group failed rapidly, whereas the fiber-reinforced specimens continued to bear load. In addition, fibers with an appropriate length increased the crack width on the fracture surface, indicating that the fibers participated in the flexural load-bearing process. The fracture surfaces of the specimens appeared white, and no obvious protruding fibers were observed. The fracture surfaces of the fiber-reinforced groups were slightly darker than those of the control group, indicating that basalt fibers, LRS, and the weak alkaline solution jointly formed white gel polymers, thereby improving the flexural performance. When the fiber length was 18 mm, more pores appeared on the fracture surface, indicating that excessively long fibers affected the formation of the internal load-bearing structure and adversely affected the mechanical properties of the LRS geopolymer.

**Figure 9:**
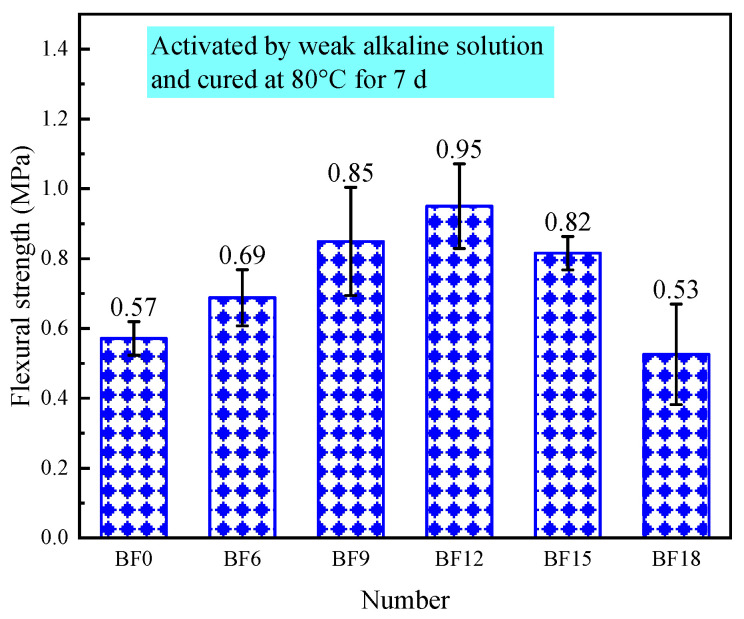
Effect of different fiber lengths on flexural strength of LRS geopolymers after curing for 7 d.

Under weak alkali activation and curing at 80 °C for 7 d, the flexural strength of LRS geopolymers with different fiber lengths is shown in Figure 9. The flexural strength ranged from 0.53 to 0.95 MPa. Compared with the control group, the incorporation of an appropriate amount of basalt fibers improved the flexural strength of the LRS geopolymers. When the fiber lengths were 6, 9, 12, and 15 mm, the flexural strength increased by 21.1%, 49.1%, 66.7%, and 43.9%, respectively. As the basalt fiber length increased, the flexural strength first increased and then decreased, indicating that fibers with an appropriate length significantly improved the flexural performance of LRS geopolymers, whereas excessively long fibers had an adverse effect.

### 3.2. Stress–Strain Behavior

#### 3.2.1. Stress–Strain Curve

**Figure 10:**
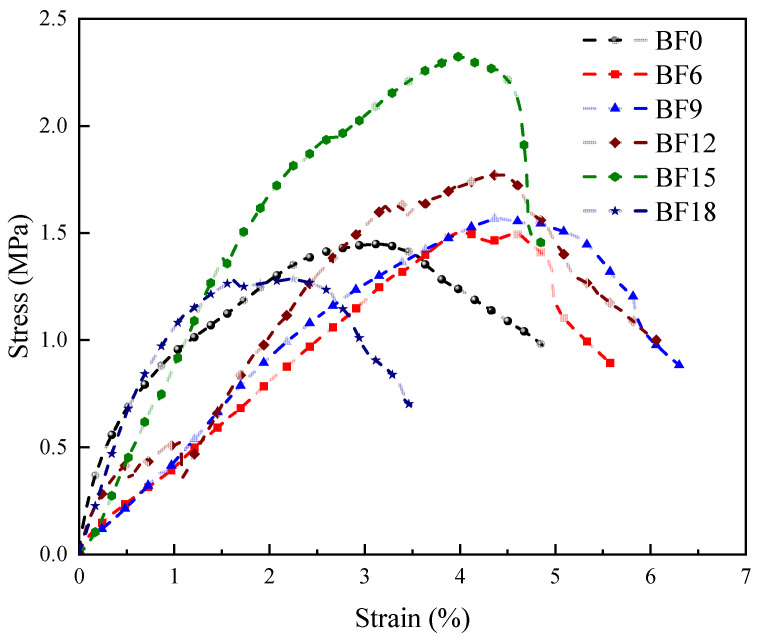
Effect of different fiber lengths on stress–strain curves of LRS geopolymers after curing for 1 d.

Under weak alkali activation and curing at 80 °C for 1 d, the stress–strain curves of LRS geopolymers with different fiber lengths are shown in Figure 10. Without fibers, the curve was generally flat, indicating ductile failure characteristics. After 6–15 mm basalt fibers were incorporated, the curve opening gradually narrowed, the yield point became more obvious, and the peak stress gradually increased. The peak position shifted backward compared with that of the control group, but gradually shifted forward as fiber length increased, indicating that fibers with an appropriate length improved the overall stress and strain levels of the geopolymer. When 18 mm basalt fibers were incorporated, the curve peak decreased, the curve opening continued to narrow, and both the stress and strain levels were weakened.

**Figure 11:**
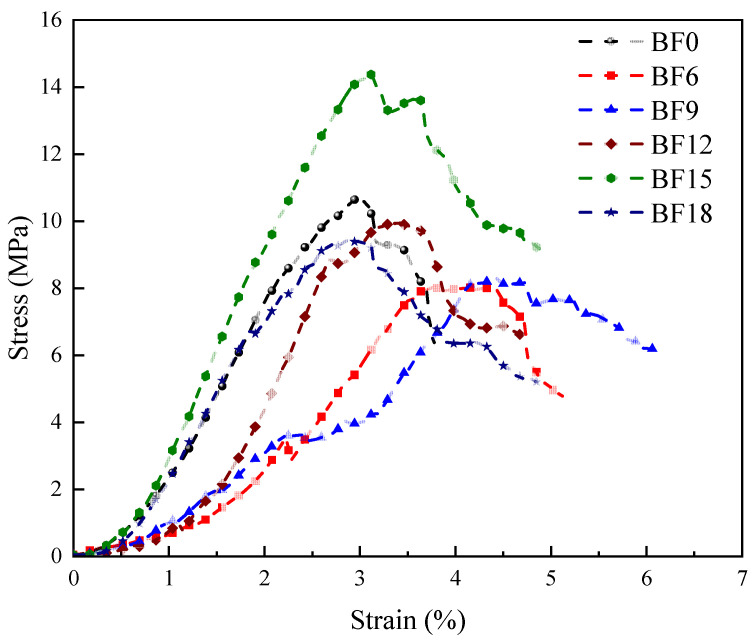
Effect of different fiber lengths on stress–strain curves of LRS geopolymers after curing for 7 d.

Under weak alkali activation and curing at 80 °C for 7 d, the stress–strain curves of LRS geopolymers with different fiber lengths are shown in Figure 11. The curves of all groups were generally slender, and the yield points were relatively obvious. After basalt fibers were incorporated, a certain plateau region appeared after the peak point and yield point, indicating that the specimens still maintained a certain load-bearing capacity. As the basalt fiber length increased, the peak point of the curve first shifted backward and then shifted forward, while the peak strength first decreased and then increased. When the fiber length was appropriate, such as 15 mm, the ultimate strength, strain capacity, and elastic modulus of the geopolymer were improved, and the envelope area of the stress–strain curve increased. This indicated that basalt fibers with an appropriate length improved the mechanical properties and toughness of LRS geopolymers.

#### 3.2.2. Stress–Strain Model

**Figure 12:**
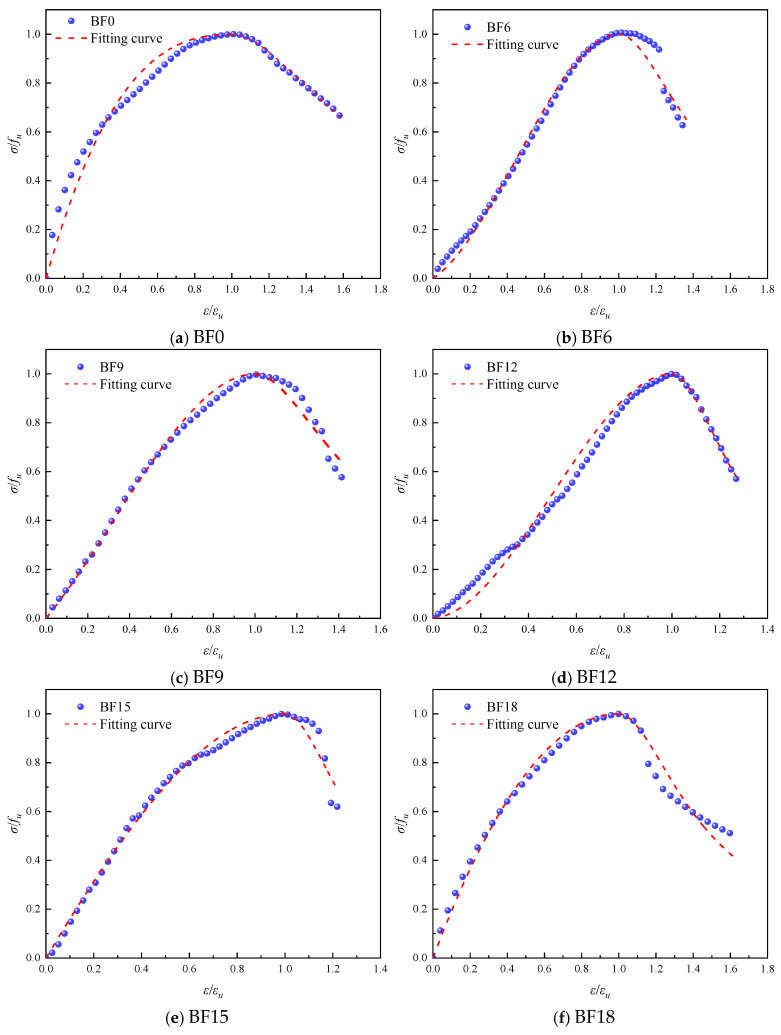
Comparison of fitting curves obtained using the Zhenhai Guo model after curing for 1 d.

**Figure 13:**
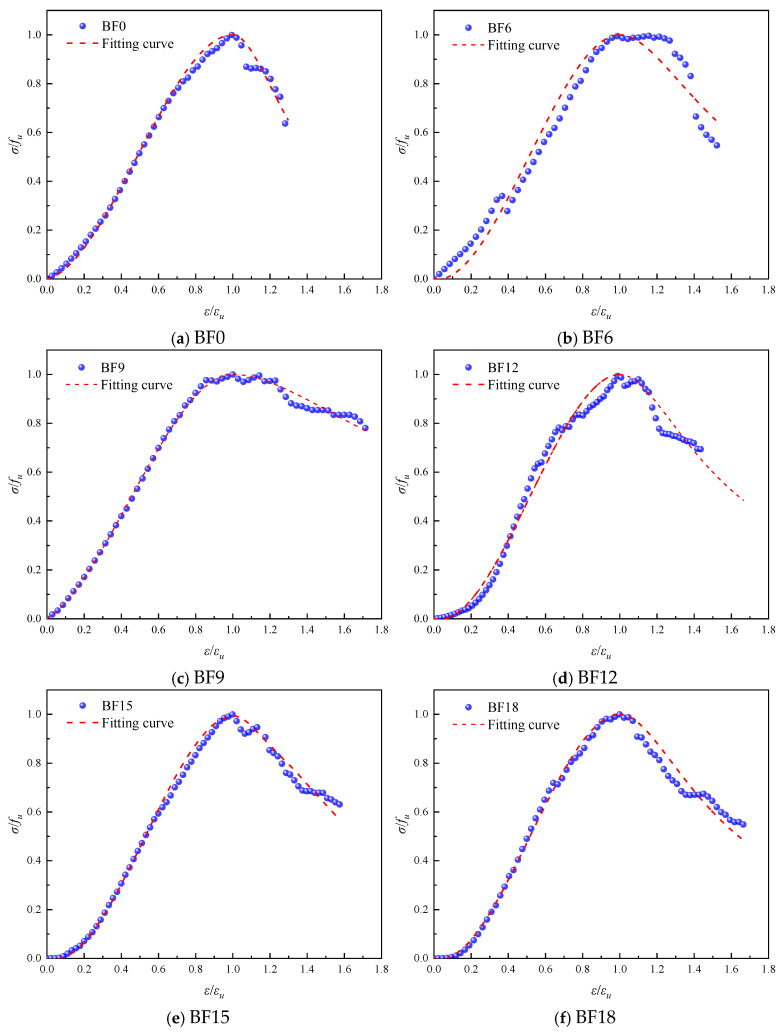
Comparison of fitting curves obtained using the Zhenhai Guo model after curing for 7 d.

**Figure 14:**
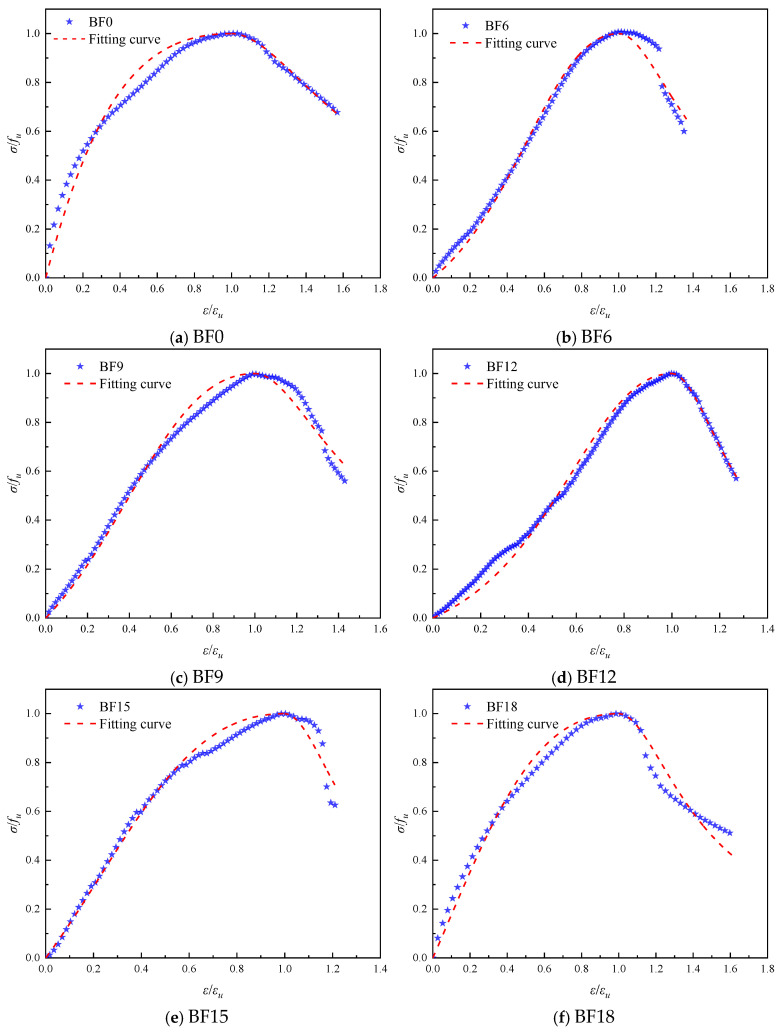
Comparison of fitting curves obtained using the Saenz L.P. model after curing for 1 d.

**Figure 15:**
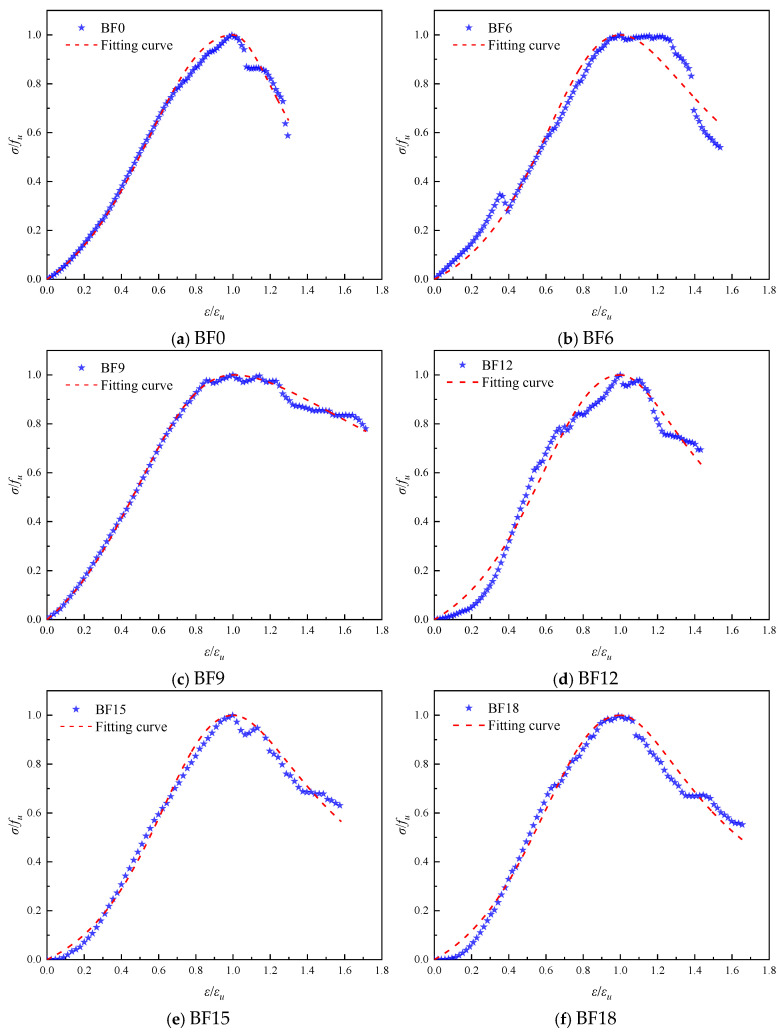
Comparison of fitting curves obtained using the Saenz L.P. model after curing for 7 d.

**Figure 16:**
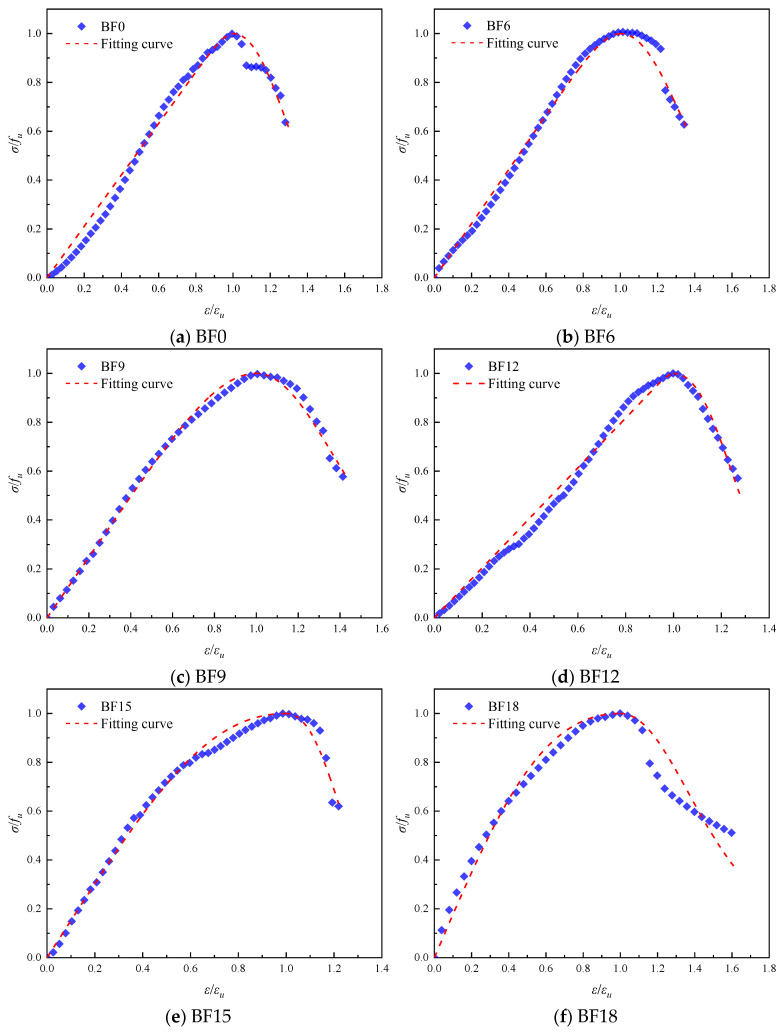
Comparison of fitting curves obtained using the Carreira D.J. model after curing for 1 d.

**Figure 17:**
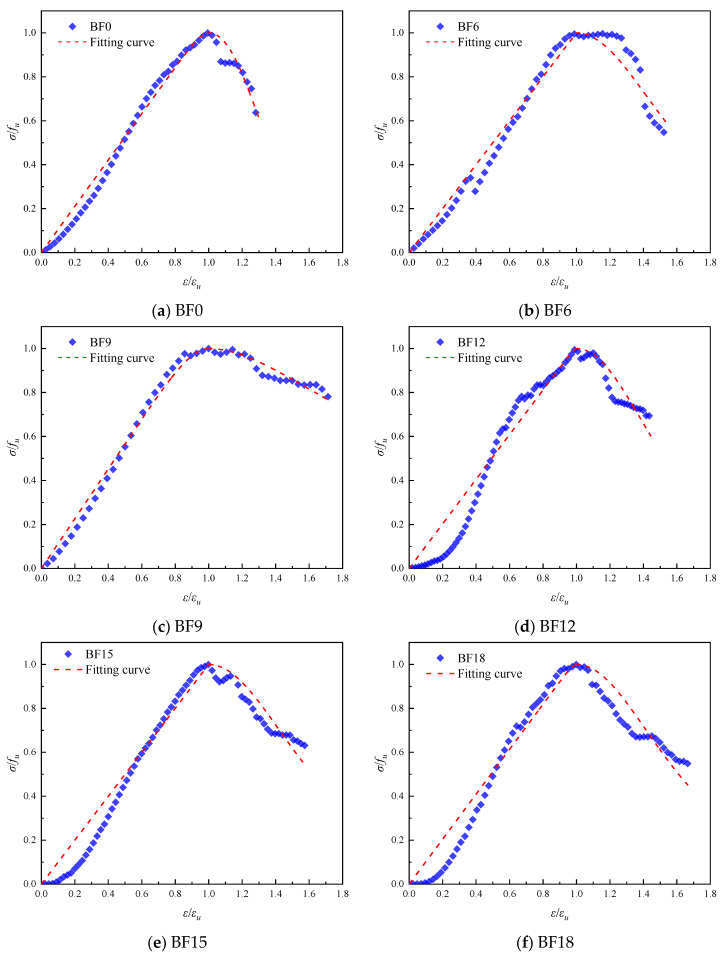
Comparison of fitting curves obtained using the Carreira D.J. model after curing for 7 d.

To establish a theoretical description of the stress–strain relationship of the geopolymer, several constitutive models widely used for ordinary concrete were selected as references, including the Zhenhai Guo model [25], the Saenz L.P. model [26], and the Carreira D.J. model [27]. Their specific expressions are listed in Table 4. Subsequently, the experimentally obtained stress–strain curves were normalized, and the above models were used to fit the ascending and descending branches of the LRS geopolymer curves with different fiber lengths. The corresponding fitting parameters and results are presented in Table 5. The comparisons between the normalized experimental curves and the theoretical fitting curves for each group are shown in Figure 12, Figure 13, Figure 14, Figure 15, Figure 16 and Figure 17.

**Figure 18:**
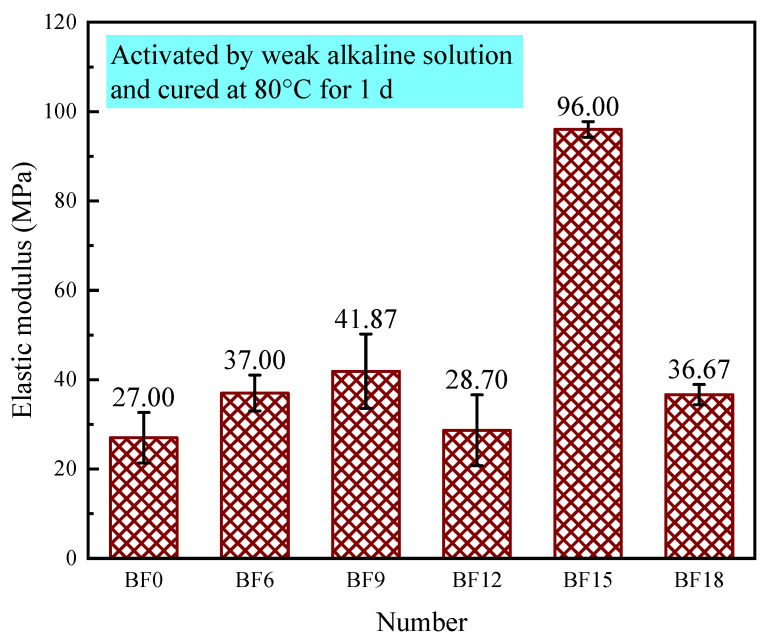
Effect of different fiber lengths on elastic modulus of LRS geopolymers after curing for 1 d.

According to the fitting results in Table 5, the correlation coefficients (*R*^2^) of the three models showed the same trend for both the ascending and descending branches of the stress–strain curves: the Zhenhai Guo model had the highest *R*^2^, followed by the Saenz L.P. model, while the Carreira D.J. model had relatively lower *R*^2^ values. This indicated that, among the selected models, the Zhenhai Guo model provided a better description of the stress–strain relationship of alkali-activated basalt fiber-reinforced LRS geopolymers. The comparisons between the experimental curves and theoretical fitting curves in Figure 13, Figure 14, Figure 15, Figure 16, Figure 17 and Figure 18 also show that, under different basalt fiber lengths, the Zhenhai Guo model better reflected the variation characteristics of the stress–strain curves of LRS geopolymers in both the ascending and descending branches and achieved the highest fitting accuracy.

Based on the comprehensive experimental results and statistical analysis, the reference values of the peak strain of basalt fiber-reinforced LRS geopolymers were obtained, as shown in Table 6.

#### 3.2.3. Elastic Modulus

Based on the stress–strain curves of the LRS geopolymers, the elastic modulus was obtained by linear fitting in the strain range of 0.01–0.015, as shown in Figure 18. After basalt fibers were incorporated, the elastic modulus of the LRS geopolymers increased, indicating improved stiffness and a higher early-age strength growth rate. When the basalt fiber lengths were 6, 9, 12, 15, and 18 mm, the elastic modulus of the LRS geopolymers increased by 37%, 55%, 6%, 255%, and 36%, respectively. The elastic modulus reached its peak when the basalt fiber length was 15 mm, increasing approximately 2.5-fold compared with the control group. This indicated that, under weak alkali activation and curing at 80 °C for 1 d, 15 mm basalt fibers greatly improved the stiffness and hardness of the geopolymer. When the fiber length was 18 mm, the elastic modulus decreased sharply, indicating that excessively long basalt fibers reduced the elastic modulus of the LRS geopolymer under weak alkali activation and high-temperature curing conditions.

**Figure 19:**
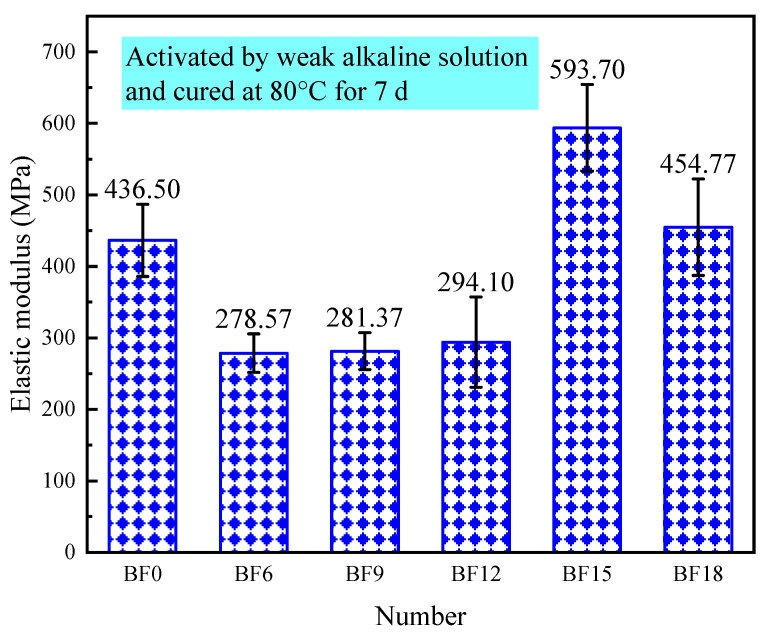
Effect of different fiber lengths on elastic modulus of LRS geopolymers after curing for 7 d.

Based on the stress–strain curves of the LRS geopolymers, the elastic modulus was obtained by linear fitting in the strain range of 0.01–0.015, as shown in Figure 19. After basalt fibers were incorporated, the elastic modulus of the geopolymers increased, indicating that their stiffness and early-age strength growth rate were enhanced. When the fiber lengths were 6, 9, 12, 15, and 18 mm, the elastic modulus increased by 37%, 55%, 6%, 255%, and 36%, respectively. Among them, the 15 mm fiber group reached the highest elastic modulus, which was approximately 2.5 times higher than that of the control group, indicating that this fiber length significantly improved the stiffness of the geopolymer. However, when the fiber length increased to 18 mm, the elastic modulus decreased markedly, suggesting that excessively long fibers weakened the stiffening effect of basalt fibers on the geopolymer.

#### 3.2.4. Peak Strain

**Figure 20:**
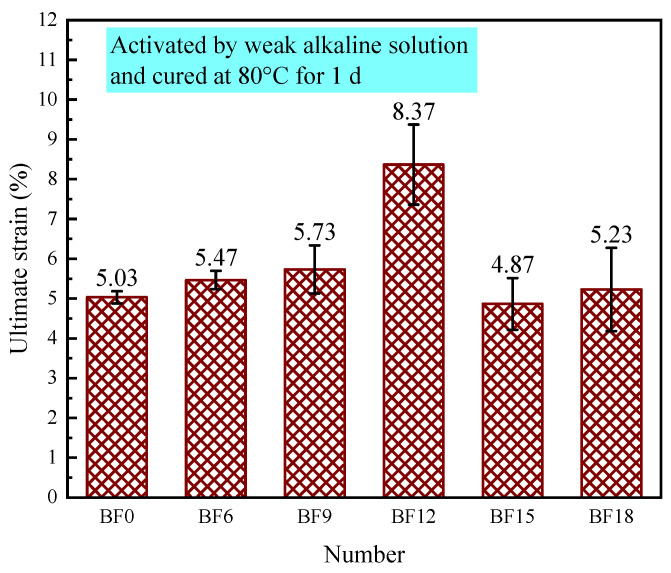
Effect of different fiber lengths on ultimate strain of LRS geopolymers after curing for 1 d.

Under weak alkali activation and curing at 80 °C for 1 d, the ultimate strains of LRS geopolymers with different fiber lengths are shown in Figure 20. The ultimate strains of all groups showed only slight differences and ranged from 4.87% to 8.37%. Among them, the 12 mm fiber group exhibited the largest increase in ultimate strain, which was 66% higher than that of the control group. Although the ultimate strain changed only slightly, the compressive and flexural strengths of the geopolymer were significantly improved after basalt fibers were incorporated. This indicates that the fibers enabled the specimens to maintain a certain load-bearing capacity after matrix failure, thereby improving the ductility of the geopolymer.

**Figure 21:**
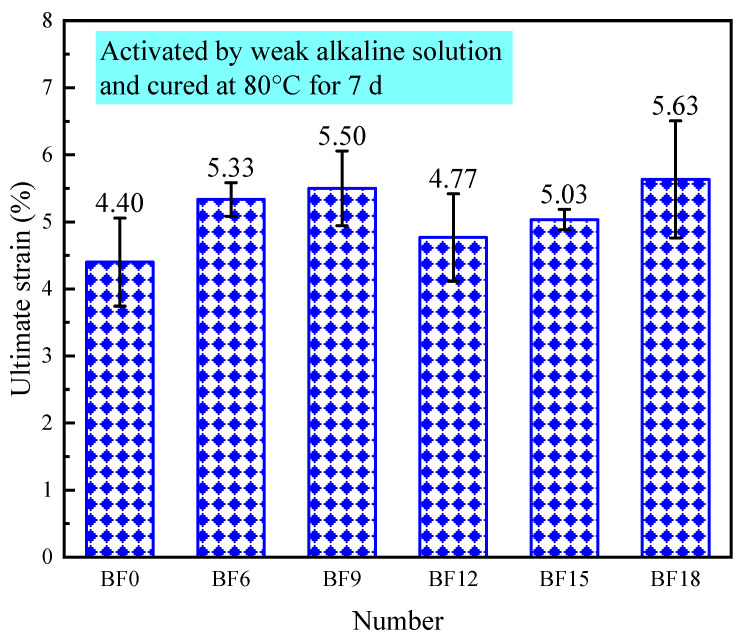
Effect of different fiber lengths on ultimate strain of LRS geopolymers after curing for 7 d.

Under weak alkali activation and curing at 80 °C for 7 d, the ultimate strains of LRS geopolymers with different fiber lengths are shown in Figure 21. The ultimate strains of all groups showed only slight differences and ranged from 4.40% to 5.63%. The overall values were relatively low, indicating that the failure mode of the specimens tended to be brittle. Among them, the 18 mm fiber group exhibited the largest increase in ultimate strain, which was 28.0% higher than that of the control group. Although the ultimate strain changed only slightly, the compressive and flexural strengths of the LRS geopolymers were significantly improved after basalt fibers were incorporated, and the specimens still maintained a certain load-bearing capacity after reaching the peak strain of the control group. This indicated that basalt fibers improved the ductility and post-peak load-bearing capacity of the LRS geopolymers.

### 3.3. SEM-EDS Analysis

**Figure 22:**
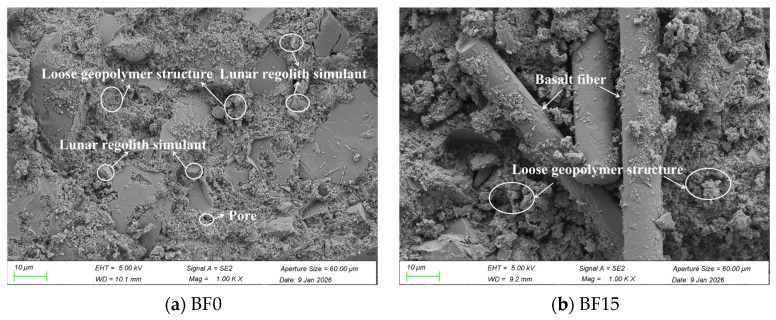
SEM images of different LRS geopolymers.

As shown in Figure 22a, the internal structure of the LRS geopolymer without basalt fiber was relatively loose. A large number of incompletely reacted lunar regolith simulant particles, pores, and loose geopolymer gel structures were distributed in the matrix. The connections between particles were not sufficiently compact, and obvious cavities and weak interfaces were locally present, indicating that the geopolymer reaction products did not fully fill the interparticle voids and that the overall structural continuity was poor. Under external loading, these pores and loose regions easily caused stress concentration, which allowed cracks to propagate rapidly along particle interfaces or pores, thereby resulting in the low load-bearing capacity of the specimens. As shown in Figure 22b, after 0.1% basalt fiber was incorporated, obvious fibers were observed in the matrix, and geopolymer reaction products adhered to the fiber surfaces and surrounding areas, indicating that a certain interfacial bond formed among the basalt fibers, LRS particles, and geopolymer gel. The fibers played bridging, tying, and crack-arresting roles in the matrix, connecting the dispersed particles and gel structures and improving the overall integrity of the material. When microcracks formed under loading, the fibers spanned both sides of the cracks, restrained crack opening and propagation, and dissipated part of the failure energy through fiber pull-out, interfacial friction, and fiber stretching. Therefore, after basalt fibers were incorporated, the internal load-bearing structure of the LRS geopolymer became more stable, crack propagation was inhibited, and the compressive strength, flexural strength, and ductility were all improved.

**Figure 23:**
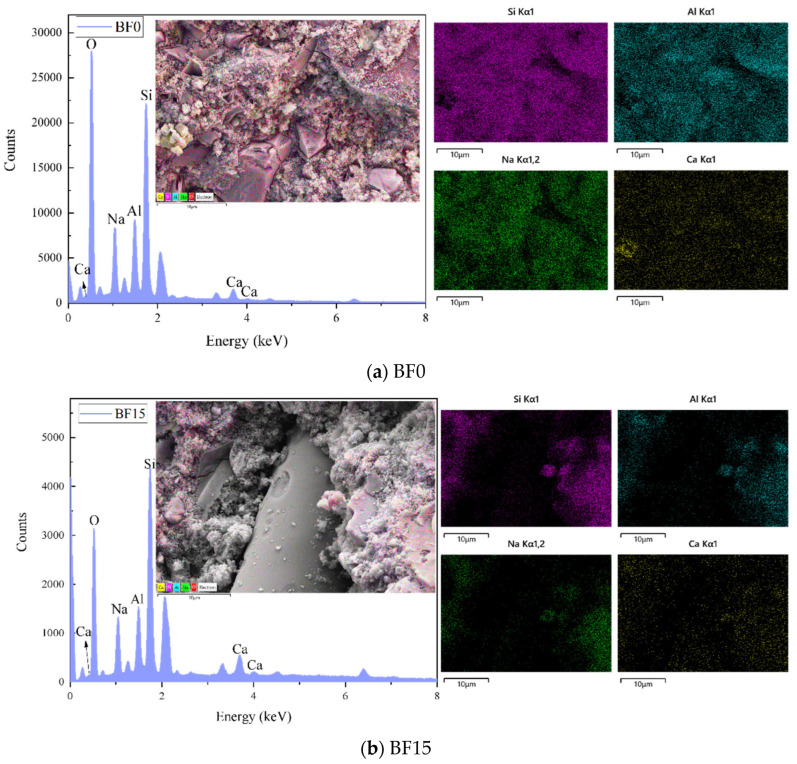
EDS images of different LRS geopolymers.

According to the SEM-EDS results shown in Figure 23, obvious characteristic peaks of O, Si, Al, Na, and Ca were detected in all LRS geopolymer specimens, and no other abnormal elements were observed. This indicates that the LRS raw material is a natural pozzolanic material rich in Si and Al, which can react with the alkali to form an amorphous three-dimensional aluminosilicate network gel. As shown in Figure 23, the Si peak had the highest intensity, whereas the Al, Na, and Ca peaks were relatively low. After 0.1% basalt fiber was incorporated, the EDS results showed that the Si/Al, Na/Si, and Ca/Si ratios of the LRS geopolymer increased. This was because basalt fibers contained certain amounts of SiO_2_ and CaO. Under weak alkali activation and high-temperature curing conditions, the fiber surface may have released some reactive Si and Ca components, which participated in the geopolymerization reaction and increased the relative contents of Si and Ca in the system. Meanwhile, basalt fibers could serve as attachment and nucleation sites for gel products, promoting the enrichment of N-A-S-H, C-A-S-H, or C-(N)-A-S-H gels near the fiber–matrix interface. Na^+^ could balance the negative charge of the [AlO_4_]^−^ tetrahedra and participate in the formation of the gel structure. Therefore, fiber incorporation not only provided physical reinforcement but also improved the gel structure and elemental distribution in the interfacial zone, making the internal structure of the LRS geopolymer more stable and thereby enhancing its mechanical properties.

## 4. Discussions

Based on the macroscopic mechanical properties and microstructural analysis, the reinforcing effect of basalt fibers on LRS geopolymers did not depend solely on fiber incorporation but was closely related to fiber length. Basalt fibers with an appropriate length formed effective bridging, tying, and crack-arresting effects in the geopolymer matrix, thereby improving the compressive performance, flexural performance, and deformation capacity of the specimens. When the fibers were too short, their embedment depth and effective anchorage length were insufficient to fully bridge cracks and transfer loads, resulting in a limited reinforcing effect on the matrix. In contrast, when the fibers were too long, they were more likely to bend, entangle, or disperse unevenly during mixing, which increased local pores and weak interfaces and weakened the continuity of the internal load-bearing structure of the LRS geopolymer.

From the macroscopic test results, the 15 mm basalt fibers showed the best performance in terms of compressive strength and elastic modulus. After curing at 80 °C for 1 d, the compressive strength of the 15 mm fiber group reached 2.23 MPa, which was 49.7% higher than that of the control group, and its elastic modulus also reached the peak value, increasing approximately 2.5-fold compared with the control group. This indicated that fibers with this length significantly improved the load-bearing capacity and stiffness of the geopolymer. After curing at 80 °C for 7 d, the compressive strength of the 15 mm fiber group reached 13.44 MPa, which was 32.0% higher than that of the control group, and its elastic modulus increased to 593.7 MPa, representing an increase of 36.0% compared with the control group. This also showed a good reinforcing effect. Although specimens with other fiber lengths achieved higher flexural strength at certain curing ages, the 15 mm fiber group exhibited more balanced performance in compressive strength, flexural strength, elastic modulus, stress–strain curve shape, and post-failure load-bearing capacity. Therefore, it had the best overall mechanical performance. From the failure modes and stress–strain curves, the 15 mm basalt fibers effectively improved the crack development process of the specimens. Compared with the specimens without fibers, the specimens reinforced with fibers of an appropriate length exhibited restrained crack propagation and still maintained a certain residual load-bearing capacity after failure. The peak stress increased, and the envelope area of the stress–strain curve became larger, indicating that the energy absorption capacity and toughness of the material were improved. This showed that 15 mm fibers had sufficient length to bridge microcracks and also maintained good dispersion in the matrix, allowing them to continuously exert a bridging effect during crack initiation and propagation.

The microstructural analysis further explained the above macroscopic performance changes. The SEM results showed that the LRS geopolymer without fibers contained many unreacted particles, pores, and loose gel structures, and the connections between particles were weak. These defects easily caused stress concentration under external loading and induced crack propagation. After 0.1% basalt fibers were incorporated, geopolymer reaction products adhered to the fiber surfaces and formed a certain interfacial bond with LRS particles and the gel matrix, connecting the originally dispersed particles and gel structures and improving the overall integrity of the matrix. The EDS results showed that the Si/Al, Na/Si, and Ca/Si ratios increased after fiber incorporation, indicating that basalt fibers may have provided some reactive Si and Ca components and promoted the enrichment of N-A-S-H, C-A-S-H, or C-(N)-A-S-H gels at the fiber–matrix interface, which was beneficial for forming a more stable interfacial transition zone. Therefore, 15 mm basalt fibers became the optimal fiber length because they achieved a good balance between effective bridging length and good dispersion. Fibers with this length fully bridged and restrained cracks, enhanced the interfacial bonding and load transfer between the fibers and the LRS geopolymer matrix, and did not cause obvious agglomeration, increased porosity, or structural defects like excessively long fibers. Macroscopically, this was reflected in significant improvements in compressive strength, elastic modulus, and toughness. Microscopically, it was reflected in the enrichment of interfacial gel products, and enhanced structural continuity. Therefore, under the conditions of weak alkali activation, high-temperature curing at 80 °C, and a fiber content of 0.1%, 15 mm was considered the optimal basalt fiber length for reinforced LRS geopolymers in this study. The improvement in strength and ductility can be attributed to the combined effects of fiber bridging, crack deflection, and interfacial anchorage. When the fiber length was appropriate, such as 15 mm, the fibers could effectively bridge microcracks and delay unstable crack propagation, thereby improving compressive strength, elastic modulus, and post-peak load-bearing capacity. However, excessively short fibers provided insufficient anchorage length, while excessively long fibers tended to disperse poorly and introduce additional pores or weak zones. Therefore, the reinforcing effect of basalt fibers in LRS geopolymers was controlled by the balance between effective crack bridging and defect formation.

## 5. Conclusions

In this study, CQU-1 LRS was used as the matrix material, and the effects of basalt fiber length on the mechanical properties, failure modes, stress–strain behavior, and microstructure of LRS geopolymers were investigated under weak alkali activation and high-temperature curing at 80 °C. The main conclusions were as follows:

(1) Basalt fiber length had a significant effect on the mechanical properties of LRS geopolymers. Basalt fibers with an appropriate length improved the compressive strength, flexural strength, and elastic modulus of the geopolymers and enhanced their post-peak load-bearing capacity. Fibers that were too short could not fully exert their bridging and crack-arresting effects, whereas excessively long fibers tended to cause poor dispersion, increased porosity, and local structural defects, thereby weakening the reinforcing effect.
(2) Based on the compressive strength, flexural strength, elastic modulus, ultimate strain, and stress–strain curve characteristics, 15 mm was identified as the optimal basalt fiber length for reinforced LRS geopolymers under the conditions used in this study. After curing at 80 °C for 1 d, the compressive strength of the 15 mm fiber group reached 2.23 MPa, which was 49.7% higher than that of the control group, and the elastic modulus was approximately 2.5 times higher than that of the control group. After curing at 80 °C for 7 d, the compressive strength of the 15 mm fiber group reached 13.44 MPa, which was 32.0% higher than that of the control group, and the elastic modulus increased to 593.7 MPa, representing an increase of 36.0%. These results indicated that 15 mm fibers achieved a favorable balance between effective bridging length and good dispersion.
(3) Curing age had an important effect on the performance development of basalt fiber-reinforced LRS geopolymers. Compared with curing at 80 °C for 1 d, curing for 7 d increased the compressive strength, flexural strength, and elastic modulus of the geopolymers, indicating that prolonged high-temperature curing promoted the continuous alkali activation reaction and the formation of gel products. However, as curing age increased, the stiffness of the specimens increased, the failure mode gradually changed from ductile to brittle, and the deformation capacity decreased.
(4) The failure modes and stress–strain curves showed that basalt fibers effectively restricted crack propagation and improved the residual load-bearing capacity of the specimens after failure. After fibers with an appropriate length were incorporated, crack development was restrained, the peak stress increased, the yield point and post-peak plateau became more obvious, and the envelope area of the stress–strain curve increased. This indicated that basalt fibers improved the toughness and energy dissipation capacity of LRS geopolymers, with the 15 mm fiber group showing better overall load-bearing behavior.
(5) Microstructural analysis showed that the unreinforced LRS geopolymer contained many unreacted particles, pores, and loose gel structures, which easily caused stress concentration and induced crack propagation under loading. After basalt fibers were incorporated, geopolymer gel products adhered to the fiber surfaces and formed good interfacial bonding with the LRS particles and matrix, providing bridging, tying, and crack-arresting effects. The EDS results showed that the Si/Al, Na/Si, and Ca/Si ratios increased after fiber incorporation, which promoted the enrichment of gel products in the interfacial zone. Based on the macroscopic and microscopic results, 15 mm was recommended as the optimal basalt fiber length for LRS geopolymers under the conditions used in this study.

It should also be noted that the mechanical properties obtained in this study are still much lower than those required for conventional structural construction. Therefore, the present work should be regarded as an exploratory study on fiber reinforcement and constitutive behavior of LRS geopolymers, rather than a direct demonstration of structural-grade lunar construction materials. From an application perspective, the improvement in strength, ductility, and post-peak load-bearing capacity caused by appropriate basalt fiber length may provide useful guidance for the design of lunar in situ construction materials. Such materials may be considered for lunar roads, landing pads, protective barriers, habitat module protective layers, or non-load-bearing prefabricated components. However, the mechanical strength obtained in this study is still far below that required for conventional structural construction. Therefore, the present results should be regarded as a preliminary reference for material optimization and mechanical modeling, rather than direct evidence for immediate structural application.

### Limitations and Future Work

Although this study investigated the effect of basalt fiber length on the mechanical behavior, constitutive response, and microstructure of CQU-1 LRS geopolymers, several limitations should be noted. First, CQU-1 lunar regolith simulant was used instead of actual lunar regolith. Differences in mineralogical composition, glassy phase content, particle morphology, and alkaline activation reactivity may exist between the simulant and real lunar regolith. Second, only short curing ages of 1 d and 7 d were considered, and the long-term strength development, shrinkage behavior, and durability of the materials remain unclear. Third, the basalt fiber content was fixed at 0.1%, and the coupled effects of fiber content and fiber length were not investigated. Fourth, the specimens were prepared and tested under terrestrial laboratory conditions, which did not reproduce the lunar environment, including vacuum, radiation, extreme thermal cycling, and reduced gravity. Fifth, although three parallel specimens were prepared for each condition, the total number of specimens was still limited. Future studies should include actual lunar regolith or more representative simulants, longer curing ages, different fiber contents, simulated lunar environmental conditions, and a larger sample size to improve the reliability and engineering relevance of the results.

The relatively low mechanical strength obtained in this study may be partly related to the limited amount of reactive glassy phases in CQU-1 LRS, the high proportion of crystalline minerals, and the weak alkali activation condition used. Future work should quantify the glassy phase content of CQU-1 LRS and compare it with real lunar regolith using XRD–Rietveld refinement, selective dissolution, FTIR, and NMR.

To facilitate practical implementation, future research should focus on improving the reactivity of LRS precursors, optimizing alkaline activator composition, investigating the coupled effects of fiber length and fiber content, and evaluating the performance of LRS geopolymers under simulated lunar environmental conditions, including vacuum, thermal cycling, radiation, and reduced gravity. Component-level tests should also be conducted for landing pads, road panels, shielding blocks, and modular construction units.

## Figures and Tables

**Table 1:** Chemical component of real lunar regolith and LRSs (wt%) [1].

Oxides	SiO_2_	TiO_2_	Al_2_O_3_	Fe_2_O_3_	CaO	MgO	Na_2_O	K_2_O	P_2_O_5_
CQU-1 [13]	45.31	2.80	15.01	15.67	8.34	3.41	4.50	3.33	0.65
BH-1 [9]	43.30	2.90	16.50	16.70	8.80	3.00	3.80	3.30	0.70
HUST-1 [19]	48.23	2.96	18.29	11.19	7.89	4.41	3.70	2.15	0.50
Chang’E-5 [17]	42.20	5.00	10.80	22.50	11.00	6.48	0.26	0.19	0.23
Apollo 14 [18]	48.10	1.70	17.40	10.40	10.70	9.40	0.70	0.55	0.51

**Table 2:** Physical properties of basalt fibers.

Monofilament Diameter	Density (g/cm^3^)	Elastic Modulus (GPa)	Tensile Strength (MPa)	Service Temperature	Bonding Temperature
10 μm	2.63~2.65	91~110	3000~4800	−269–650 °C	1050 °C

**Table 3:** Proportion of LRS geopolymer.

Number	Fiber Length (mm)	Water/Binder Ratio	Modulus	Na_2_O (%)	LRS (g)	Na_2_SiO_3_ (g)
BF0	0	0.456	3.3	8.3	100	100
BF6	3				100	100
BF9	6				100	100
BF12	9				100	100
BF15	12				100	100
BF18	15				100	100

**Table 4:** Full curve model of concrete stress–strain.

Model	Equation	Coefficient	
Saenz L.P. model [26]	$y=\frac{mx}{1+x(m-2)+x^{2}}$	*m*	(2)
Carreira D.J. model [27]	$y=\frac{mx}{m-1+x^{m}}$	*m*	(3)
Zhenhai Guo model [25]	$y=mx+(3-2m)x^{2}+(m-2)x^{3},0\leq x<1$ $y=\frac{x}{m{\left(x-1\right)}^{2}+x},x\geq 1$	*m*	(4)

Note: *y = σ*/*f_u_*; *σ* is stress; *f_u_* is peak stress; *x = ε*/*ε_u_*; *ε* is the strain; *ε_u_* is the peak strain.

**Table 5:** Fitting values of the shape coefficient for the LRS geopolymer stress–strain curve model.

**Model**	**Order of Curve**	**Fiber Length (mm)**		**0**		**6**		**9**	
			**Age (d)**	**1**	**7**	**1**	**7**	**1**	**7**
		**Coefficient**							
Saenz L.P. model [26]	Rising section	*m*		2.893	0.513	0.610	0.381	0.889	0.634
		*R* ^2^		0.950	0.995	0.994	0.983	0.987	0.999
	Falling section	*m*		0.419	0.127	0.181	0.327	0.215	0.992
		*R* ^2^		0.993	0.804	0.856	0.775	0.909	0.862
Carreira D.J. model [27]	Rising section	*m*		1.634	19.732	10.186	3.279	5.130	8.579
		*R* ^2^		0.941	0.984	0.994	0.977	0.996	0.989
	Falling section	*m*		4.348	9.269	7.621	5.188	6.650	2.751
		*R* ^2^		0.972	0.742	0.914	0.838	0.963	0.839
Zhenhai Guo model [25]	Rising section	*m*		2.682	0.179	0.489	−0.131	1.045	0.498
		*R* ^2^		0.949	0.997	0.996	0.968	0.995	0.999
	Falling section	*m*		2.382	7.839	5.529	3.058	4.657	1.008
		*R* ^2^		0.993	0.805	0.857	0.778	0.909	0.862
**Model**	**Order of Curve**	**Fiber Length (mm)**		**12**		**15**		**18**	
			**Age (d)**	**1**	**7**	**1**	**7**	**1**	**7**
		**Coefficient**							
Saenz L.P. model [26]	Rising section	*m*		0.442	0.441	1.314	0.365	1.730	0.425
		*R* ^2^		0.987	0.977	0.991	0.993	0.981	0.991
	Falling section	*m*		0.078	0.227	0.087	0.277	0.167	0.249
		*R* ^2^		0.998	0.856	0.768	0.892	0.882	0.869
Carreira D.J. model [27]	Rising section	*m*		47.457	74.668	2.929	9.236	2.293	43.967
		*R* ^2^		0.986	0.935	0.993	0.953	0.984	0.950
	Falling section	*m*		12.166	6.157	12.982	5.287	6.551	5.368
		*R* ^2^		0.966	0.748	0.861	0.788	0.696	0.718
Zhenhai Guo model [25]	Rising section	*m*		0.069	−0.205	1.635	−0.371	2.036	−0.212
		*R* ^2^		0.979	0.986	0.995	0.994	0.993	0.998
	Falling section	*m*		12.732	4.391	11.372	3.606	5.983	4.005
		*R* ^2^		0.998	0.856	0.769	0.892	0.882	0.869

**Table 6:** Reference value of peak strain.

	Fiber Length (mm)	0	6	9	12	15	18
Age (d)							
1		0.031	0.041	0.043	0.063	0.036	0.025
7		0.035	0.032	0.031	0.03	0.031	0.035

## Data Availability

The original contributions presented in this study are included in the article. Further inquiries can be directed to the corresponding author.
